# Supplementary material for: Exon-junction complex association with stalled ribosomes and slow translation-independent disassembly
Source: Nat Commun. 2024 May 17;15:4209. doi: 10.1038/s41467-024-48371-5 (PMC11101648; doi:10.1038/s41467-024-48371-5)
Supplement: Supplementary file 1 — Supplementary Information [file 41467_2024_48371_MOESM1_ESM.pdf]

Supplementary Information List

Supplementary Figure S1 –
Transcripts immunoprecipitated with EJCs.

Supplementary Figure S2 –
Persistence of EJC binding is transcript specific.

Supplementary Table 1
Centrosome, kinetochore and microtubule linked genes

Supplementary Table 2.
Chemicals

Supplementary Table 3.
Immunological reagents

Supplementary Table 4.
Plasmids

Supplementary Table 5.
Cells

Supplementary Table 6.
Software and algorithms

Supplementary Table 7.
Apparatuses

Supplementary Methods 1
Numerical analysis of recovery kinetics after DRB removal

Supplementary references

Supplementary Figure 1

Exon-junction complex association with stalled ribosomes and slow translation-independent disassembly

a

Mitochondrial transcripts (RPM)

	DRB	0h00	1h00	2h00
Replicate I	Ab	27504	59947	93498
	blk	146677	125649	144968
	Inp	133268	150543	148337
Replicate II	Ab	53461	42913	59801
	blk	115535	103964	105887
	Inp	82337	83372	70266

e

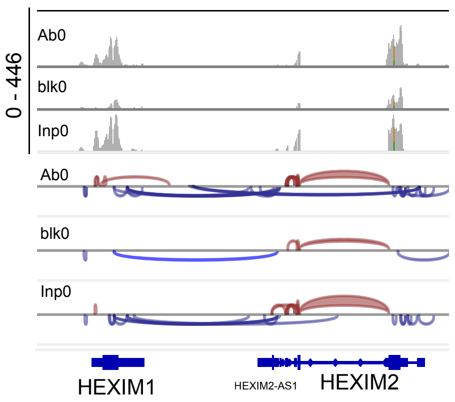

b

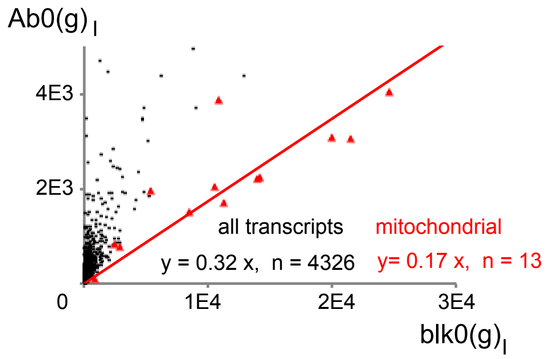

c

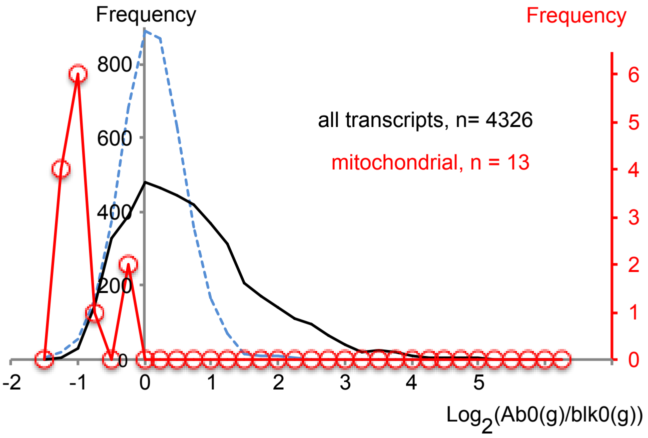

f

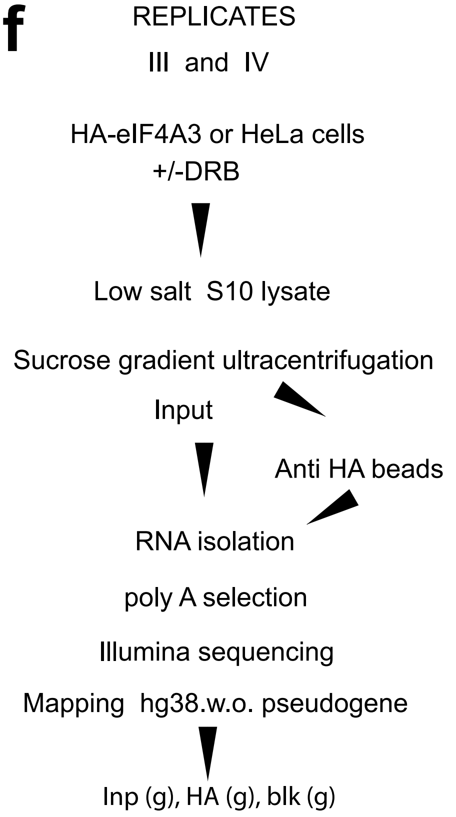

d

Normalization factors, α

	DRB	0H00	1H00	2H00
Replicate I	α	0.17	0.46	0.60
Replicate II	α	0.45	0.41	0.55

g **h** **i**

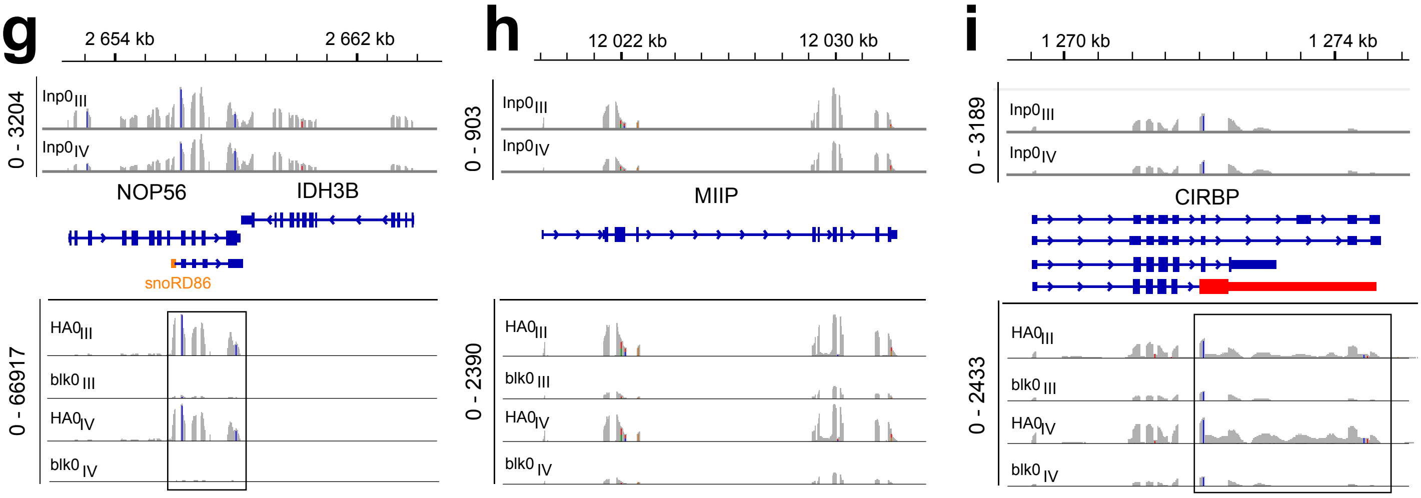

Supplementary Figure 1 - Transcripts immunoprecipitated with EJCs. (a) Total mitochondrial encoded reads immunoprecipitated (Ab), blank precipitated (blk) and input (Inp) expressed in reads per million (RPM) in replicates I and II. (b) Immunoprecipitated reads ($Ab0(g)_I$) are plotted versus blank precipitated reads ($blk0(g)_I$) for replicate I. Red triangles correspond to mitochondrial-coded transcripts (Supplemental Data 3). (c) Frequencies of enrichment within 0.25 intervals for $Log_2(blk0(g)_I/blk0(g)_{II}$ (dotted blue curve) or $Log_2(Ab0(g)/blk0(g)_{II}$ (black curve – all genes, red curve – mitochondrial genes). (d) Summary of calculated of normalization factors a. (e) IGV profiles for the intron-less *HEXIM1* gene (replicate I). Shashimi plots are shown below. (F) Pipeline for RNA isolation and sequencing of replicates III and IV; (g – i) IGV profiles for *NOP56*, *MIIP* and *CIRBP* genes for replicates III and IV. The *snoRD86* in *NOP56* transcript is in orange. The deficient splicing of *CIRBP* is underlined in red. Read scales are shown on the left of each profiles. Reads immunoprecipitated with antibodies (HA0) or without (blk0), and inputs (Inp0) are grouped separately.
!

Supplementary Figure 2

Exon Junction Complex association with stalled ribosomes and slow translation-independent disassembly

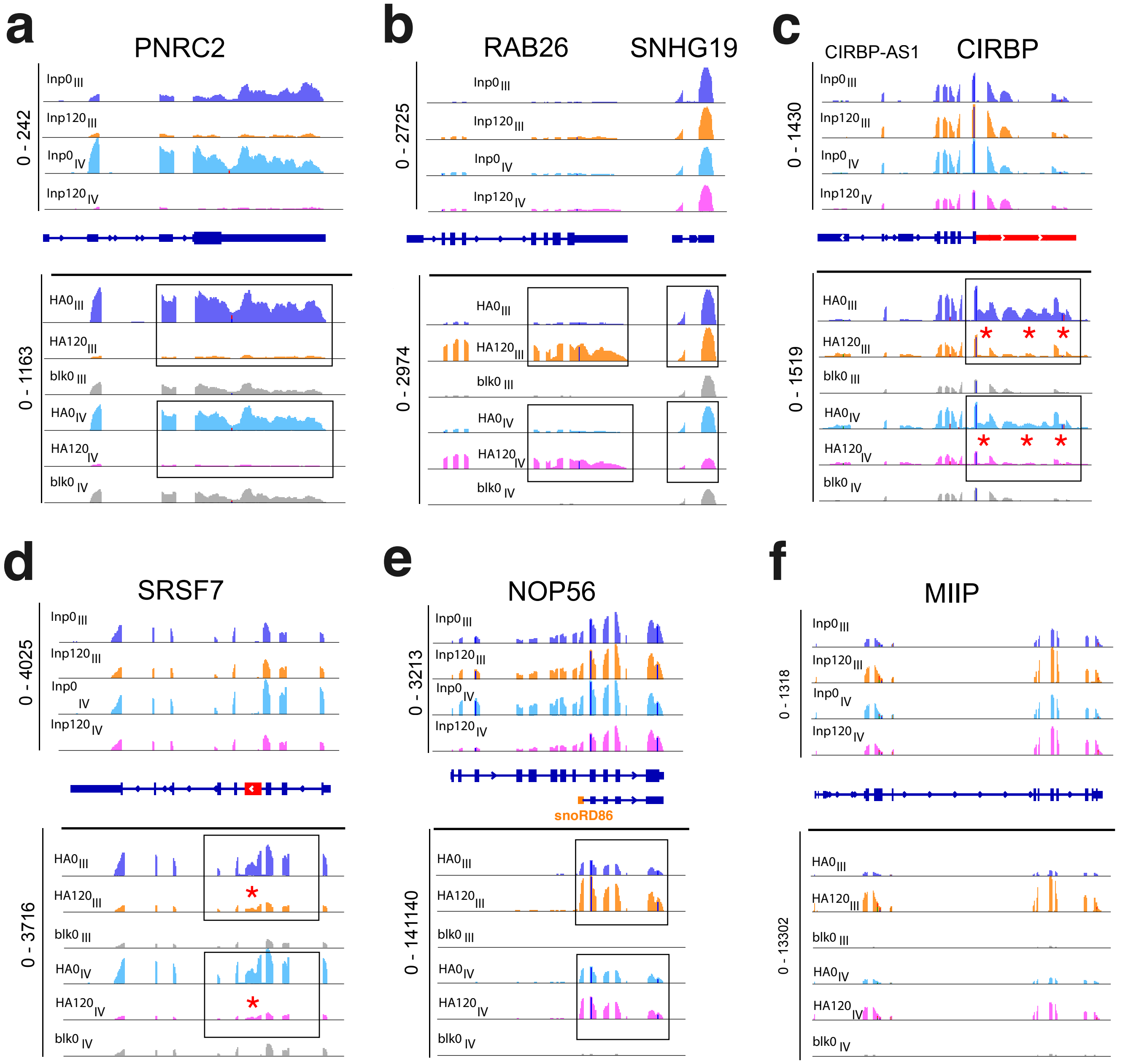

Supplementary Figure 2 - Persistence of EJC binding is transcript specific. (a – f) IGV profiles without DRB (blue) or following 2H00 DRB treatment (orange) are shown for the same genes than Figure 6 for replicates III and IV. No treatment (blue) or 2h00 DRB treatment (orange or pink). Blank precipitated reads are in grey. Red stars marks reads specifically enriched in immunoprecipitates. Read scales are shown on the left of each profiles. Reads immunoprecipitated with antibodies (HA0) or without (blk0), and inputs (Inp0) are grouped separately.

Supplementary Table 1 – Centrosome, kinetochore and microtubule linked genes found in the 546 more than 4-fold EJC-enriched transcripts, ranked by decreasing lifetimes. Top ones are the most persistent ones. Ranking numbers are shown on the right column. Data from Supplemental Data file 6.

Name 1	Name 2	Function	Source and or reference	Rank
FOPNL	CEP20	Located in centriolar satellite and nucleoplasm. Involved in cilium assembly.	UniProt	7
MIIP	IIp45	binds to and inhibits HDAC6 that deacetylates alpha-tubulin. regulates the mitotic checkpoint	Reference 1, 2	8
PIAS1		Sumoylates ninein. switches ninein localization from centrosome to nucleus	Reference 3	12
CHEK2	CHK2	Inhibits microtubule nucleation ; regulates centrosome maturation.	Uniprot, & Reference 4	13
IQCB1	NPHP5	Involved in ciliogenesis. The function in an early step in cilia formation depends on its association with CEP290/NPHP6	Uniprot	15
FANCD2		FA-mutant cells exhibited mitotic spindle anomalies such as supernumerary centrosomes and shorter mitotic spindles.	Reference 5	16
TUBE1		Tubulin Epsilon 1	Uniprot	18
UBAP2L		necessary for correct kinetochore-microtubule attachment.	Reference 6	22
KIAA0753		CEP120-mediated recruitment onto centrioles. regulates ciliogenesis	Reference 7, 8	25
TROAP	TASTIN	required for bipolar spindle assembly and centrosome integrity during mitosis. TROAP is in a pathway downstream of EZH2.	Reference 9	26
KANSL1		stabilizes microtubule minus ends in a RanGTP-dependent manner, essential for spindle assembly and chromosome segregation.	Reference 10	28
CFAP97		Cilia- and flagella-associated protein 97	Uniprot	30
DNMBP		Dynamin-binding protein, associated with the actin and microtubule cytoskeleton ; regulates spindle orientation.	Uniprot	36
ASPM		regulates microtubule dynamics at spindle poles	Uniprot	57
CUL9		Ubiquitin-protein ligase complex. required to maintain microtubule dynamics	UniProt	58
EZH2		colocalizes with the microtubule organization center (MTOC). EZH2 is in a pathway upstream of TROAP	Reference 11, 12	61
ARHGEF 4	ASEF	guanine nucleotide exchange factor. Asef interacted with APC and associated with microtubule fraction upon HGF stimulation	Reference 13	62
KIF18B		constitutes the major microtubule plus-end depolymerizing activity in mitotic cells	Uniprot	64
KMT5A	SET8	Binds α-tubulin and methylates it at a specific lysine residue	Reference 14	65
GAS8		Dynein regulatory complex subunit 4	Uniprot	69
SFI1	A8K8P3	Plays a role in the dynamic structure of centrosome-associated contractile fibers via its interaction with CETN2	Uniprot	75
WDR90		Cilium biogenesis/degradation – centrosome scaffold	Uniprot & Reference 15	80
PKD1		is activated and associated with centrosomes, spindles, and midbodies.	Reference 16	89
AHCTF1	ELYS MEL28	Nucleoporin. Interacts with the γ-tubulin ring complex and recruits it to microtubule nucleation sites	Reference 17	100
TXLNG		required for centriolar subdistal appendage assembly and microtubule organization	Reference 18	104
CEP95		Centrosomal protein of 95 kDa	Uniprot	107
ESPL1		Extra spindle poles-like 1 protein	Uniprot	126
UBR2		Mub1/Ubr2 ubiquitin ligase complex associates with kinetochore particles	Reference 19	127
HAUS8		Contributes to mitotic spindle assembly, maintenance of centrosome integrity and completion of cytokinesis	Uniprot	134
FANCA		interacts with γ-tubulin and localized to centrosomes, most notably during the mitotic phase,	Reference 20	150
POGZ		Knockdown of POGZ causes defects in sister-chromatid cohesion and kinetochore protein assembly.	Reference 21	152
TUBGCP 6		Gamma-tubulin complex necessary for microtubule nucleation at the centrosome	Uniprot	157
DNHD1		Dynein heavy chain domain-containing protein 1 - microtubule binding motor protein	Uniprot	159
CNTROB		Centriole Duplication And Spindle Assembly Protein	Uniprot	175
UBR5		E3 ubiquitin ligase UBR5 regulates centriolar satellite stability and primary cilia	Reference 22	178
USP21		USP21 as a regulator of centrosome- and microtubule-associated functions	Reference 23	180
CCDC78		Component of the deuterosome, a structure that promotes de novo centriole amplification in multiciliated cells	Uniprot	186
KAT2A	GCN5	mediates acetylation of α-tubulin	Reference 24	190

BIRC6		Important regulator for the final stages of cytokinesis.	Uniprot	207
**ALMS1**		Centrosome-associated protein	Uniprot	217
OFD1		Component of the centrioles controlling mother and daughter centrioles length. Involved in the biogenesis of the cilium	Uniprot	217
PDE4D		NDE1, NDEL1 and LIS1, together with their binding partner dynein, associate with DISC1, PDE4B and PDE4D and this complex is present at the centrosome.	Reference 25	231
ATXN7		Necessary for microtubule cytoskeleton stabilization	Uniprot	238
CCDC57		Pleiotropic regulator of centriole duplication, mitosis, and ciliogenesis. Critical interface between centrosome and microtubule-mediated cellular processes.	Uniprot	242
KNTC1		Required for the assembly of the dynein-dynactin and MAD1-MAD2 complexes onto kinetochores	Uniprot	252
PCNT		Integral component of the filamentous matrix of the centrosome	Uniprot	255
KIF20B		Kinesin-like protein KIF20B	Uniprot	256
CEP72		Centrosomal protein	Uniprot	263
KIFC2		Kinesin-like protein KIFC2	Uniprot	270
NAT9		Alpha/beta-tubulin-N-acetyltransferase 9, colocalizes with mitotic spindles	Uniprot & Reference 26	284
TPR		acts as a spatial regulator of the spindle-assembly checkpoint (SAC) response ensuring a timely and effective recruitment of spindle checkpoint proteins to kinetochore during the metaphase-anaphase	Uniprot	285
IFT43		Involved involved in ciliogenesis ; retrograde ciliary transport along microtubules	Uniprot	287
BCL2L11		BCL2L11 recruits BECN1 to microtubules by bridging BECN1 and DYNLL1	Reference 27	297
ANKRD26		interacts with and recruit the PIDDosome component PIDD1 to centriole distal appendages,s	Reference 28	298
CENPT		Centromere protein T	Uniprot	311
DYNC1LI 2		Cytoplasmic dynein 1 light intermediate chain 2	Uniprot	334
C7orf43	TRAPPCC	mediates RAB3IP preciliary vesicle trafficking to the mother centriole during ciliogenesis	Uniprot	339
SYNE2		Required for centrosome migration to the apical cell surface during early ciliogenesis.	Uniprot	348
WDR60	DYNC2I1	Cytoplasmic dynein 2 intermediate chain 1	Uniprot	351
CROCC	Rootletin	Major structural component of the ciliary rootlet, Contributes to centrosome cohesion before mitosis	Uniprot	355
NINL		Ninein-like protein, interferes with mitotic spindle assembly.	Uniprot	360
NEURL4		maintains normal centriolar homeostasis and preventing formation of ectopic microtubular organizing centers	Uniprot	363
CFAP53		Cilia- and flagella-associated protein 53	Uniprot	364
CEP295		Centrosomal protein	Uniprot	370
TUBG2		Tubulin gamma-2 chain	Uniprot	392
AKAP9	AKAP450/CG-NAP/AKAP9	Scaffolding protein that assembles several protein kinases and phosphatases on the centrosome and Golgi apparatus. Required for association of the centrosomes with the poles of the bipolar mitotic spindle during metaphase.	Uniprot	431
LUZP1		actin and microtubule-associated protein, which localizes to the centrosome ; restricts primary cilia formation	Reference 29	434
NAIP		accumulates in the mitotic spindle poles and is shown in spindle microtubules	Reference 30	439
ZNF207	BuGZ	Kinetochore- and microtubule-binding protein that plays a key role in spindle assembly	Uniprot	475
CBX5		Involved in the formation of functional kinetochore	Uniprot	519

Supplementary Table 2 – Chemicals

	Source	Identifier
Zeocin	Invivogen	ant-zn-5
Neomycin, G418	InvivoGen	ant-gn-5
puromycin	InvivoGen,	ant-pr-1
Actinomycin D	Sigma	A9415-2MG
Cycloheximide	Toku-E,	SKU: C084-1ML
Harringtonine	Biorbyt	# orb322316
5,6-Dichloro-1-b-D-ribofuranosylbenzimidazole (DRB)	Sigma	D1916-10MG
JET Prime	Polyplus	#114-15
Passive Lysis Buffer	Promega	E1941
Furimazine	AOUBIOUS	#AOB36539-5
RNase inhibitor	Promega	#N2611
Protease inhibitor cocktail	Calbiochem	#535140
RNase A/T1 mix	Thermo Scientific	#EN0551
SYBR green	BioVision	#B1747-5
Trizol	Thermofisher Scientific	#15596018

Supplementary Table 3 – Immunological reagents

Antibodies	Source		Identifier
Anti HA	Sigma	WB 1/5000 dilution	H6908
Anti Flag	Sigma	WB 1/5000 dilution	F7425
eIF4A3	Affinity purified [31]	WB 1/1000 dilution	#3360
Y14/RBM8A	Affinity purified [32]	WB 1/1000 dilution	#3210
MAGOH	Gift E. Izaurralde [33]	WB 1/1000 dilution	-
GAPDH	Cell Signaling Technology	WB 1/1000	14C10
Anti-rabbit HRP	Promega	WB 1/5000	W401B
Anti-mouse HRP	Promega	WB 1/5000	W402B
Clean-Blot™ IP Detection Reagent (HRP)	Thermofisher		#21230
ECL Western Blotting Substrate	Pierce™		Kit 34580
Protein A Dynabeads	Thermofisher		#10008D
Anti HA magnetic beads	Life Technologies		#88837

Supplementary Table 4 – Plasmids

Plasmids	Source	Identifier
pBiT1.1-C [TK/LgBiT]	Promega	N2014
pBiT2.1-C [TK/SmBiT]	Promega	N2014
pBiT2.1-N [TK/SmBiT]	Promega	N2014
CMV MAGOH-C-LgBiT Neo^{Res}	MAGOH cDNA, LgBiT from pBiT1.1-C [TK/LgBiT) inserted into pcDNA3	pHLH1692
CMV eIF4A3-C-SmBiT Puro^{Res}	eIF4A3 cDNA, SmBiT from pBiT2.1-C [TK/SmBiT) inserted into pcDNA3 where the neomycin gene has been replaced by puromycin resistance gene	pHLH1696
CMV Flag-eIF4A3-C-SmBiT Puro^{Res}	Flag-tag inserted N-terminus of eIF4A3 in CMV eIF4A3-C-SmBiT Puro^{Res}	pHLH1723
CMV HA-MAGOH-C-LgBiT Neo^{Res}	HA-tag inserted N-terminus of MAGOH	pHLH1736

	in CMV MAGOH-C-LgBiT Neo^{Res}	
CMV HA-MAGOH-C-LgBiT KND/A Neo^{Res}	mutagenesis of CMV HA-MAGOH-C-LgBiT Neo^{Res}	pHLH1744
CMV Flag eIF4A3-C SmBiT Zeo^{Res}	Zeocin^{Res} replaces Puro^{Res} in CMV Flag-eIF4A3-C-SmBiT Puro^{Res}	pHLH1775
CMV Flag-eIF4A3-C-SmBiT D401K E402R	mutagenesis of CMV Flag-eIF4A3-C-SmBiT Puro^{Res}	pHLH1785
CMV HA-N-SmBiT-MAGOH	HA-SmBiT from pBiT2.1-N [TK/SmBiT] fused N-terminal of MAGOH cDNA in pCDNA3	pHLH1791
CMV Flag-Y14 C-LgBiT Zeo^{Res}	Y14 cDNA C-terminal LgBiT replaces eIF4A3 cDNA and C-terminalSmBiT in CMV Flag eIF4A3-C SmBiT Zeo^{Res}	pHLH1792

Supplementary Table 5 – Cells

Cells	Source	Identifier
HA-eIF4A3	From HeLa cells, HA tag inserted by CRISPR in all eIF4A3 alleles described in [34]	-
HeLa	Genuine HeLa cells used in [34]	
HEK293 Trex	invitrogen #R71007	R71007
OB2 cells express eIF4A3-SmBiT	HEK293 Trex^{TM} transfected with pHLH1696, puromycin selection	OB2
OB3 cells express MAGOH-LgBiT	HEK293 Trex^{TM} transfected with pHLH1692, neomycin selection	OB3
OB9 cells express Flag-eIF4A3-SmBiT/MAGOH-LgBiT,	HEK293 Trex^{TM} transfected with pHLH1692 and pHLH1723, zeocin and neomycin selection	OB9
OB20 cells express Flag-eIF4A3-SmBiT/HA-MAGOH-LgBiT,	HEK293 Trex^{TM} transfected with pHLH1723 and pHLH1736, neomycin and puromycin selection	OB20

Supplementary Table 6 – Software and algorithms

	Source	Identifier
HISAT2	Galaxy France	Version 2.2.1+galaxy1
Featurecounts v2.3	Galaxy France	Version 2.0.3+galaxy1
Integrative Genomics Viewer	https://igv.js	Version 2.16.0
Amigo gene ontology	https://amigo.geneontology.org/amigo	

Supplementary Table 7 – Apparatuses

	Source	Identifier
Fluorimeter	INFINITE M NANO* TECAN	microplate reader
Gradient collector	ISCO	model UA6
Luminometer	Berthold	TriStar LB941
Fragment Analyzer	Agilent	
Q-Bit	Invitrogen	Q-Bit -3
Rotor	BECKMAN	SW41

Supplementary Methods 1

Numerical analysis of recovery kinetics after DRB removal

At steady state

$d(EJC)/dt = R_{ass}^{TD} + R_{ass}^{TinD} - R_{diss}^{0\text{-}TD} - R_{diss}^{0\text{-}TinD} = 0$ (1)

Assembly rates (R_{ass})= disassembly rates (R_{diss})

$R_{ass}^{TD} + R_{ass}^{TinD} = R_{diss}^{0\text{-}TD} + R_{diss}^{0\text{-}TinD}$ (2)

At steady state ($EJC^{0\text{-}TinD}$) = concentration of EJC disassembled by translation dependent (TD) mechanism; concentration of EJC disassembled by translation independent (TinD) mechanism.

$R_{diss}^{0\text{-}TinD} = k_{diss}^{TinD} (EJC^{0\text{-}TinD})$ and $R_{diss}^{0\text{-}TD} = k_{diss}^{TD} (EJC^{0\text{-}TD})$ (3)

As summarized in Figure 4c,

$(EJC^{TD}) = 0.3\ (EJC^{0})$; $(EJC^{TinD}) = 0.7\ (EJC^{0})$ (4)

and $k_{diss}^{TD} = 17\ k_{diss}^{TinD}$

Therefore : $R_{diss}^{0\text{-}TD} = 0.3\ k_{diss}^{TD} (EJC^{0})$ (5)

$R_{diss}^{0\text{-}TD} + R_{diss}^{0\text{-}TinD} = 17\text{x}0.3\ k_{diss}^{TinD} (EJC^{0}) + 0.7\ k_{diss}^{TinD} (EJC^{0})$ (6)

Therefore :

$$\mathbf{R_{ass}^{TD} + R_{ass}^{TinD} = 5.4\ k_{diss}^{TinD} (EJC^{0})} \quad (7)$$

In the presence of DRB

$R_{ass}^{TD} = R_{ass}^{TinD} = 0$ - No transcription hence no EJC assembly. (8)

Therefore :

$d(EJC)/dt = - R_{diss}^{0\text{-}TD} - R_{diss}^{0\text{-}TinD}$ (9)

$d(EJC)/dt = - k_{diss}^{TD} (EJC^{TD}) - k_{diss}^{TinD} (EJC^{TinD}) = - 17\ k_{diss}^{TD} (EJC^{TD}) - k_{diss}^{TinD} (EJC^{TinD})$ (10)

Rates upon removal of DRB in the presence of cycloheximide

$d(EJC)/dt = R_{ass}^{TD} + R_{ass}^{TinD} - R_{diss}^{TinD}$ because $R_{diss}^{TD} = 0$ (11)

For dissociation rates, we make calculations for (EJC) = 0.5 (EJC^{0}) after 2H00 in DRB and (EJC) = 0.75 (EJC^{0}) after 20' of recovery (Figure 4f)

$\mathbf{d(EJC)/dt} = 5.4\ k_{diss}^{TinD} (EJC^{0}) – 0.75\ k_{diss}^{TinD} (EJC^{0}) = \mathbf{4.45\ k_{diss}^{TinD} (EJC^{0})}$ (12)

Rates upon removal of DRB without cycloheximide

$d(EJC)/dt = R_{ass}^{TD} + R_{ass}^{TinD} - R_{diss}^{TD} - R_{diss}^{TinD}$ (13)

We assume that R_{ass}^{TD} and R_{ass}^{TinD} remain the same that at steady state

One can make numerical calculations for (EJC) = 0.5 (EJC^{0}) after 2H00 in DRB and (EJC) = 0.75 (EJC^{0}) after 75' of recovery (Figure 4f)

Since assembly is 7 fold higher for translation-dependent than independent EJCs and (EJC^{TD}) = 0 when DRB is removed.

It is deduced that (EJC^{TD}) = 0.21 (EJC^{0}) and (EJC^{TinD}) = 0.54 (EJC^{0})

$R_{diss}^{TD} + R_{diss}^{TinD} = 0.21\text{x}17\ k_{diss}^{TinD} (EJC^{0}) + 0.54\ k_{diss}^{TinD} (EJC^{0}) = 4.1\ k_{diss}^{TinD} (EJC^{0})$ (14)

$\mathbf{d(EJC)/dt} = 5.4\ k_{diss}^{TinD} (EJC^{0}) - 4.1\ k_{diss}^{TinD} (EJC^{0}) = \mathbf{1.3\ k_{diss}^{TinD} (EJC^{0})}$ (15)

Combining (12) and (15) :

(Rate + Cycloheximide)/(Rate without) = 4.45/1.3 **= 3.4** (16)

Supplementary references

1. Ji P*, et al.* Inhibition of gliomagenesis and attenuation of mitotic transition by MIIP. *Oncogene* **29**, 3501-3508 (2010).

2. Wang Y, Wen J, Zhang W. MIIP, a cytoskeleton regulator that blocks cell migration and invasion, delays mitosis, and suppresses tumorogenesis. *Current protein & peptide science* **12**, 68-73 (2011).

3. Cheng TS, Chang LK, Howng SL, Lu PJ, Lee CI, Hong YR. SUMO-1 modification of centrosomal protein hNinein promotes hNinein nuclear localization. *Life sciences* **78**, 1114-1120 (2006).

4. Nai S*, et al.* Chk2-dependent phosphorylation of myosin phosphatase targeting subunit 1 (MYPT1) regulates centrosome maturation. *Cell cycle (Georgetown, Tex)* **18**, 2651-2659 (2019).

5. Magron A, Elowe S, Carreau M. The Fanconi Anemia C Protein Binds to and Regulates Stathmin-1 Phosphorylation. *PloS one* **10**, e0140612 (2015).

6. Maeda M*, et al.* Arginine methylation of ubiquitin-associated protein 2-like is required for the accurate distribution of chromosomes. *FASEB journal : official publication of the Federation of American Societies for Experimental Biology* **30**, 312-323 (2016).

7. Chang CH*, et al.* CEP120-mediated KIAA0753 recruitment onto centrioles is required for timely neuronal differentiation and germinal zone exit in the developing cerebellum. *Genes & development* **35**, 1445-1460 (2021).

8. Inskeep KA*, et al.* Genetic and phenotypic heterogeneity in KIAA0753-related ciliopathies. *American journal of medical genetics Part A* **188**, 104-115 (2022).

9. Yang S, Liu X, Yin Y, Fukuda MN, Zhou J. Tastin is required for bipolar spindle assembly and centrosome integrity during mitosis. *FASEB journal : official publication of the Federation of American Societies for Experimental Biology* **22**, 1960-1972 (2008).

10. Meunier S, Shvedunova M, Van Nguyen N, Avila L, Vernos I, Akhtar A. An epigenetic regulator emerges as microtubule minus-end binding and stabilizing factor in mitosis. *Nat Commun* **6**, 7889 (2015).

11. Deshayes F, Fradet M, Kaminski S, Viguier M, Frippiat JP, Ghislin S. Link between the EZH2 noncanonical pathway and microtubule organization center polarization during early T lymphopoiesis. *Sci Rep* **12**, 3655 (2022).

12. Jin L*, et al.* EZH2-TROAP Pathway Promotes Prostate Cancer Progression Via TWIST Signals. *Frontiers in oncology* **10**, 592239 (2020).

13. Higginbotham K, Tian Y, Gawlak G, Moldobaeva N, Shah A, Birukova AA. Hepatocyte growth factor triggers distinct mechanisms of Asef and Tiam1 activation to induce endothelial barrier enhancement. *Cellular signalling* **26**, 2306-2316 (2014).

14. Chin HG*, et al.* The microtubule-associated histone methyltransferase SET8, facilitated by transcription factor LSF, methylates α-tubulin. *The Journal of biological chemistry* **295**, 4748-4759 (2020).

15. Vasquez-Limeta A, Loncarek J. Human centrosome organization and function in interphase and mitosis. *Seminars in cell & developmental biology* **117**, 30-41 (2021).

16. Martínez-León E*, et al.* Protein kinase D1 inhibition interferes with mitosis progression. *Journal of cellular physiology* **234**, 20510-20519 (2019).

17. Yokoyama H*, et al.* The nucleoporin MEL-28 promotes RanGTP-dependent γ-tubulin recruitment and microtubule nucleation in mitotic spindle formation. *Nat Commun* **5**, 3270 (2014).

18. Ma D*, et al.* α-/γ-Taxilin are required for centriolar subdistal appendage assembly and microtubule organization. *eLife* **11**, (2022).

19. Akiyoshi B, Nelson CR, Duggan N, Ceto S, Ranish JA, Biggins S. The Mub1/Ubr2 ubiquitin ligase complex regulates the conserved Dsn1 kinetochore protein. *Plos Genet* **9**, e1003216 (2013).

20. Kim S*, et al.* Fanconi anemia complementation group A (FANCA) localizes to centrosomes and functions in the maintenance of centrosome integrity. *The international journal of biochemistry & cell biology* **45**, 1953-1961 (2013).

21. Nozawa RS*, et al.* Human POGZ modulates dissociation of HP1alpha from mitotic chromosome arms through Aurora B activation. *Nature cell biology* **12**, 719-727 (2010).

22. Shearer RF*, et al.* The E3 ubiquitin ligase UBR5 regulates centriolar satellite stability and primary cilia. *Mol Biol Cell* **29**, 1542-1554 (2018).

23. Urbé S, Liu H, Hayes SD, Heride C, Rigden DJ, Clague MJ. Systematic survey of deubiquitinase localization identifies USP21 as a regulator of centrosome- and microtubule-associated functions. *Mol Biol Cell* **23**, 1095-1103 (2012).

24. Ouyang C*, et al.* Deletion of Ulk1 inhibits neointima formation by enhancing KAT2A/GCN5-mediated acetylation of TUBA/α-tubulin in vivo. *Autophagy* **17**, 4305-4322 (2021).

25. Bradshaw NJ*, et al.* DISC1, PDE4B, and NDE1 at the centrosome and synapse. *Biochem Biophys Res Commun* **377**, 1091-1096 (2008).

26. Mok JW, Choi KW. Novel function of N-acetyltransferase for microtubule stability and JNK signaling in Drosophila organ development. *Proceedings of the National Academy of Sciences of the United States of America* **118**, (2021).

27. Luo S, Rubinsztein DC. BCL2L11/BIM: a novel molecular link between autophagy and apoptosis. *Autophagy* **9**, 104-105 (2013).

28. Evans LT, Anglen T, Scott P, Lukasik K, Loncarek J, Holland AJ. ANKRD26 recruits PIDD1 to centriolar distal appendages to activate the PIDDosome following centrosome amplification. *Embo J* **40**, e105106 (2021).

29. Gonçalves J. LUZP1: A new player in the actin-microtubule cross-talk. *European journal of cell biology* **101**, 151250 (2022).

30. Abadía-Molina F, Morón-Calvente V, Baird SD, Shamim F, Martín F, MacKenzie A. Neuronal apoptosis inhibitory protein (NAIP) localizes to the cytokinetic machinery during cell division. *Sci Rep* **7**, 39981 (2017).

31. Daguenet E*, et al.* Perispeckles are major assembly sites for the exon junction core complex. *Mol Biol Cell* **23**, 1765-1782 (2012).

32. Wang Z, Murigneux V, Le Hir H. Transcriptome-wide modulation of splicing by the exon junction complex. *Genome biology* **15**, 551 (2014).

33. Kwon OS*, et al.* Exon junction complex dependent mRNA localization is linked to centrosome organization during ciliogenesis. *Nat Commun* **12**, doi 10.1038/s41467-41021-21590-w (2021).

34. Hocq R, Paternina J, Alasseur Q, Genovesio A, Le Hir H. Monitored eCLIP: high accuracy mapping of RNA-protein interactions. *Nucleic Acids Res* **46**, 11553-11565 (2018).
