# Supplementary material for: Exon-junction complex association with stalled ribosomes and slow translation-independent disassembly
Source: Nat Commun. 2024 May 17;15:4209. doi: 10.1038/s41467-024-48371-5 (PMC11101648; doi:10.1038/s41467-024-48371-5)
Supplement: Supplementary file 3 — Description of Additional Supplementary Files [file 41467_2024_48371_MOESM3_ESM.pdf]

# Description of Additional Supplementary Files

**Supplementary Data 1**

**List of assigned reads number per gene** for replicate I (Page A) and replicate II (Page B) data sets (62,657 genes in the hg38.109 reference human genome) (Columns D to L). Cells are treated 0, 60 or 120 minutes with DRB. Inp0, Inp60 and Inp120 are reads from RNA-sequencing of gradient fractions (Inputs). RNAs immunoprecipitated from gradient fractions with anti-HA antibodies (Ab0, Ab60 and Ab120) or without antibodies (blank) (blk0, blk60 and blk120) from the same gradient fractions. Column M to O : RNAs immunoprecipitated from untreated cell gradient fractions are normalized for sequencing depth (reads per million) and averaged for both replicates. Columns P to X : Sequenced reads are normalized for sequencing depth and average transcript length : RPKM, reads per million per kilobase.

**Supplementary Data 2**

**Inputs expressed in RPKM** (reads per kilobase per million). (Page A) Data from Supplemental Data 1 filtered for an averaged Ab0(g) >50 RPM and corresponding to 4,326 genes. Columns K, L, M provide averaged RPKM. (Page B) Data from A filtered for Ab0(g) > 4*blk0(g) on average and corresponding to 546 genes. **Stability indexes of transcripts** (Columns N, O) defined as Inp60(g)/Inp0(g) and Inp120(g)/Inp0(g).

**Supplementary Data 3**

**Determination of Alpha normalizing coefficients using mitochondrial transcripts as invariant genes**. Mitochondrial reads are normalized for sequence depth (RPM). Immunoprecipitated mitochondrial reads are plot versus blank reads to determine alpha for each replicates. (Page A) Replicate I; (Page B) Replicate II.

**Supplementary Data 4**

**Ejc values expressed in RPKM** (reads per kilobase per million) for 4,326 genes filtered for an averaged Ab0(g) >50 RPM (column Q in yellow) (Page A). They are determined using the data sets provided in Supplemental Data 1 and alpha normalizing factors in Supplemental Data 2; (Page B) Same as A but for 3,110 genes with Ab0(g) > blk0(g); (Page C) Same as A and B but for the 546 genes with Ab0(g) > 4*blk0(g).

**Supplementary Data 5**

**Gene Ontology analysis of EJC-enriched genes**. (A) Analysis of 546 genes with Ab0(g) > 4*blk0(g) for cellular compartment using the Amigo server (B) same as A but for 3,110 genes with Ab0(g) > blk0(g). Number of genes expected (column D) is the ratio of total number of genes in the category in the genome (column B) / total number of genes in the genome with gene ontology annotation (20,589). The enrichment of a gene ontology category (column F) is the ratio Number of genes found (column

C) / Number of genes expected (column D).

**Supplementary Data 6**

**Persistence indexes**. Cells are treated 1H00 or 2H00 with DRB. Persistence indexes PsIn 60 = Ejc60(g)/Ejc0(g) and PsIn 120 = Ejc120(g)/Ejc0(g) are first determined independently for replicates I and II, next normalized by the median of ratio method and averaged (column S and column Z). Column AD provides an RNA stability index as the ratio of input reads Inp120(g)/Inp0(g). Data corresponds to the 546 genes enriched 4-fold (Ab0(g)>4*blk0(g)).
